# Subjective Exercise Experience and Group Cohesion among Chinese Participating in Square Dance: A Moderated Mediation Model of Years of Participation and Gender

**DOI:** 10.3390/ijerph191912978

**Published:** 2022-10-10

**Authors:** Peiyao Ji, Shihan Zhou, Ruohang Wang, Hongying Fan, Yan Wang

**Affiliations:** 1 School of Psychology, Beijing Sport University, Beijing 100084, China; 2 School of Art, Beijing Sport University, Beijing 100084, China

**Keywords:** Chinese square dancer, positive well-being, group environment, female–male, adherence

## Abstract

(1) Background: This study aimed to explore the relationship between years of participation, subjective exercise experience, and group cohesion among gender-specific square dance practitioners. (2) Methods: The Subjective Exercise Experience Questionnaire (SEEQ) and Group Environment Questionnaire (GEQ) were used to evaluate Subjective Exercise Experience (SEE) and group cohesion (GC). An analysis was conducted on 130 Chinese (63 males and 67 females) using multiple group analysis within a structural equation model. (3) Results: (a) The positive aspects of Subjective Exercise Experience (SEE) and Positive Well-Being (PWB), had a strongly positive effect on GC in both groups. The negative aspects of SEE, Psychological Fatigue (PF), and Psychological Distress (PD), had negative effects on GC. (b) Only for the male group was there an indirect effect of participation years on the association between SEE and GC in the model (a × b = 0.062, 95% CI [0.001, 0.181]; standard error (SE) = 0.062, *p* = 0.048). (c) The significant differences between paths coefficients were noticed in the association of years of participation with SEE (t = −2.043) and GC (t = −1.962). (4) Conclusion: Based on these results, gender differences in terms of the partial mediating role of adherence in the relationship of SEE and GC were presented for future research, fitness popularization, and society.

## 1. Introduction

### 1.1. Chinese Square Dance

Square dance is a mass fitness sport and cultural activity in China that has grown in popularity, in parallel with people’s demand for a better and healthier life [1]. It is considered to be a popular public activity in the squares and parks of Chinese cities [2]. Since its development, Chinese square dance has gradually spread globally, and is identifiable as a new image of China. Chinese Central Television estimates that over 100 million people in the country participate in square dancing every day [3]. The 2017 China Square Dance White Paper reported East China has the highest number of Generation Y dancers, with a proportion of as high as 43% or more [4]. Regarding the definition of square dance, different perceptions can be summarized as an active process of urban learning. It incubates sentiments of collectivism that overcome anonymity and strangeness, but that do not contradict the ethos of individualism in the modern city [5]. Especially in China, square dancing can be considered as a traditional cultural and sports activity; almost every night, organized by the masses spontaneously, carried out in public spaces (e.g., in the midst of or adjacent to housing units and recreation spaces, squares, parks, playgrounds, or courtyards all over China [2,6]), assisted by music and incorporating many dancing types to strengthen participants’ physical and mental health [7].

The movement and posture of square dancing are designed to be simple and entertaining. Compared with other dance forms or physical activities, the dancing forms vary from gymnastic exercises to folk dance and disco. It has been proven in studies that square dancing has physical benefits for participants [8]. Square dancing can be classified as a moderate intensity exercise according the Dietary Guidelines for Chinese Residents [6], and it is mainly participated in by middle-aged women and the elderly [3]. Compared to traditional exercise (e.g., swimming and running), square dancing is characterized by simple movements, and has frequently gained easy acceptance by many people [9]. However, as an aerobic exercise accompanied by a dance rhythm, square dancing engages the participants’ entire body, and Chernozub et al. [10] found that dance, rather than power fitness, had a greater influence on the reduction of women’s circumferential body size, decreased body fat mass, and increased the level of fitness, which is one of the priorities of this type of motor activity. There is also suggestion that dance, regardless of its style, can significantly improve muscular strength and endurance, balance, and other functional fitness attributes in older adults [11]. Another study compared the effects of Tai Chi exercise and square dancing on individual’s waist–hip index changes [12]. Additionally, there is also evidence that square dance can promote better physical health, including appearance, body fat, physical activity, and the entire body [13], immune function, life satisfaction [14], and improve balance and cardiorespiratory function [15].

Square dance is not only good for the exerciser’s body, but is also good for their mental health [16]. It is mainly reflected in the moderating effect of music on square dance participants. Music is the soul of square dance. The accompanying music of square dance is not restricted, but is mainly regulated by the rhythm of music. That is to say, square dancing participants perform to the accompaniment of its own music style, which makes specific groups (e.g., the retired, the middle-aged, and the elderly) who have the same taste show a passion for such a fitness activity. Previous studies have shown that age is related to music taste, and that people of different ages have different life goals and different reasons for listening to music [17,18,19]. Since the fact that middle-aged and retired group prefer lively music and coordinated dance movements [15], the majority of the music used for square dancing is edited with a plain soundtrack of drumbeats in similar rhythms. Often, the moves are made to be easy to follow with the music [3]. Additionally, others have studied the function of music in people’s daily life [20]. After summing up, there were seven main functions: enhancing positive emotions, enhancing negative emotions, killing time, establishing identity, promoting social interaction, nostalgia for the past, and understanding the world [17]; square dancing is also an effective way to maintain psychological awareness [21]. Through lively music and coordinated dance movements, square dancing also can engage a variety of cognitive skills, such as executive function, memory, and motor skills [22]. Furthermore, square dancing creates a rich social environment for participants to interact with their peers, enhancing interpersonal communication to maintain their social participation and eliminate loneliness, which was also a determinant of positive aging [23]. Enriching the dance experience contributes to a sense of self-efficacy and an active lifestyle [24]. Square dance, with its wide mass, easy to learn nature, and rich musicality, establishes a positive psychological environment for the participants.

### 1.2. Subjective Exercise Experience and Group Cohesion

Subjective exercise experience is the feeling or experience that is formed in a specific situation (physical exercise) [25]. It is a subjective experience with memories based on participation in exercise practice, which can enrich the exercise cognitive system of exercisers and enhance exercise decision-making power [26]. Cohesion is defined as the unity of the group, and the definition is central to understanding groups and the group processes used. Additionally, group cohesion is a central explanatory concept in the study of groups, which is defined as cohesion with a degree of group solidarity, and distinguishes between causes of such solidarity (such as attractive bonds or group pride) and indicators of cohesion. It can be used to assess members’ perceptions of the unity of their groups [27]. Among them, task cohesion is more related to deep-level, non-observable qualities such as attitudes and values, while social cohesion is associated with observable qualities such as age and physical condition [28].

Research has shown a link between subjective exercise experience and group cohesion. High levels of family cohesion, for example, can contribute to subjective well-being through high levels of self-concept, clarity, and hope [29]. The relationship between group cohesion and outcome was significantly modified by gender [30]. Among them, gender plays a very important role. Among the baseline variables, a low frequency of meeting with friends and subjective loneliness among males, and a low number of recreational sporting hobbies among females during the ages of 13–18 years old or so, were associated with lower life satisfaction in young adulthood (during 13–18 years old, it was also associated with lower life satisfaction in youth) [31]. It can be found that the effects of subjective exercise experience and group cohesion differ across genders. This was also the case where such a relationship was also tested across completely different situations, like family [32,33], neighborhood [34,35], team [36], and society [34,37].

Many scholars have obtained different research situations regarding the relationship between subjective exercise experience and group cohesion in the exercise domain. The study of the relationship between the two is reflected in many fields. The Chinese version of the International Physical Activity Questionnaire (LTPA) was used to evaluate the LTPA of 2783 elders (73.8% of the population was aged over 65 years old) in 47 communities in Shanghai, and found that both the social and physical attributes of the neighborhood was associated with LTPA, among older adults in China [37]. During 2006 to 2009, according to multiple-level Hurdle Negative Binomial models from two samples of 57,092 Dutch adults with higher levels of social cohesion compared to areas with less social cohesion, more people were physically active [34]. After conducting a Singapore Population Health Studies on 1012 subjects who underwent a 12-month health behavior exchange, health interventions within community members were found to be significant in relation to enhancing individual engagement and group cohesion [38]. (In addition, and in general), social cohesion was associated with the amount of PA [39]. Correlation studies have also emerged across exercise years and gender populations. Involvement in both leisure activity categories, but not in social activities, was significantly and positively associated with mental health status in both men and women (16,642 middle-aged Japanese adults, aged 50–59). Furthermore, in men, both “hobbies or cultural activities” and “exercise or sports” were only significantly related to mental health status only when performed “with others”. Among women, the effects of “hobbies or cultural activities” on mental health status were no different, regardless of the ways of participating, while the result of “exercise or sports” had the same results as men [40]. Higher ratings of cooperation within the group predicted a better post-intervention quality of life, and physical functioning and less fatigue in men after the intervention, while a better quality of life and physical functioning was found in females [41]. Therefore, it is worth conducting and testing the cohesion and subjective experience while undertaking physical activities. The research found that there is also a link between subjective exercise experience and group cohesion in the exercise domain. The results found that participation in square dance can be the moderator of the relationship between family cohesion and subjective well-being after investigating 331 middle-aged and empty-nest Chinese women [42].

### 1.3. Adherence to Physical Activity

Adherence is a key term when considering long-term engagement in an intervention. Within the field of exercise research, this term is often used synonymously with that of compliance, concordance, or participation [43]. In the situation of fitness-enhancing exercises [44], it refers to years of participation [16], length of participation [45], exercise prevalence [46], and so on.

Studies have found a number of links between adherence and subjective exercise experience [47]. Measuring peer-directed adherence has the potential to be a cost-effective approach when developing exercise programs for older adults [48] who improve their health and fitness through physical activity. Demographic factors, health status, physical factors, and psychological factors were associated with better adherence in older age [49].

The expectation of social interaction is a very important indicator as a positive predictor of adherence to physical activity. It is not an aim toward physical activity, while it is a means of achieving self-cohesion [50]. Linking physical activity with social practices can help people to become more physically active, and can provide better results in adherence exercise programs for the general population [51]. In the context of health-enhancing physical activity promotion, especially in exercise classes, some (observational and experimental) evidence points to the utility of shared social identities around salient characteristics, as well as social identity leadership in promoting exercise adherence [52]. However, psychometric properties of the Group Environment Questionnaire were investigated in a large sample of soccer (n = 222) and professional basketball players (n = 375). No significant associations were found between team cohesion and the duration of belonging to the team, which contrasts with expectations. The length of membership in different groups had different effects on the relationship between cohesion and the individual’s decision to participate in exercise on a regular basis [53]. The wide variability of the duration of belonging, ranging from less than one year up to 20 years, may have had a large impact on this outcome. Therefore, future cross-sectional and longitudinal studies are recommended to provide larger cohorts that allow for a deeper exploration of the role of this variable, as well as the effects of other variables, both at individual (i.e., age) and team (i.e., category) levels [54].

### 1.4. Motivation of Participating in Physical Activity

Motivation is interpreted as a journey of intrinsic incentives and extrinsic factors that cause demand [55]. Studies have found that gender differences are one of the factors that influence an individual’s motivation to participate in physical activity [56]. Gender differences are explained in the context of role expectations and self-concept development [57]. Among adults (but not among children), the profile picture of physically strong male dancers was identified as male significantly more so than the profile picture of weaker male dancers [58]. Harrington [59] referred to this phenomenon as a range of physicality that embodied different aspects of the psyche, with some being socially defined as “masculine” or “feminine” movement, or as being socially structured to adopt a physicality that is passive in nature. Furthermore, these would influence people’s preference or motivations before people know it. For example, in general, boys choose activities that develop manipulative, but also locomotor skills (basketball, handball, and soccer), while girls mostly choose to participate in activities that have great potential for development of the locomotor skills (gymnastics, swimming, and dancing) [60]. In the case of causing boys not to learn aesthetic movements at all, this could produce alienation for aesthetic movements in general. Then, the research coming out of psychology and neurobiology has supported the exploration of a diverse range of movement qualities to foster both physical and psychological fluidity between expressions by dance [59].

Motivation plays an important role; in more detail, gender characteristics lead to the concept that males’ motivations are in contrast to females’ motivations. Among the motivational characteristics assessed, the main factor associated with physical activity for girls was the “relatedness” component (positive peer relationships and inclusion/cooperative activities). In contrast, physical activity related to boys included all three motivational characteristics, specified using the self-determination theory (support for autonomy, mastery/competence, and inclusion/relevance) [61]. Likewise, a higher preference for cooperation in females relative to males was linked with a differential activation of the medial prefrontal cortex [62], and larger gray matter volumes in the bilateral posterior inferior frontal and left anterior medial prefrontal cortices [63]. To be more specific, the majority of women reported that they exercised to become or to stay fit (77.0%), with losing weight (29.6%), unwinding (22.2%), socializing (18.2%), and having fun (17.8%) following as the most common motivations among women. Among men, the most prevalent motivation to exercise was becoming or staying fit (71.9%), having fun (25.3%), unwinding (24.4%), losing weight (22.7%), and socializing (22.7%) [64]. The effect of gender on physical activity levels was mainly direct, but motivation was a significant mediator. This was demonstrated by higher levels of motivation in men compared to women, but not in participant satisfaction [65]. This article aims to investigate whether the motivation of men and women to participate in physical activity moderates the mediating variable years of participation under gender differences.

### 1.5. Hypotheses

Summing up the above research, duration, subjective exercise experience, and group cohesion are related, respectively. Additionally, gender always makes a difference for the motivations of participating in a physical activity, this being subjective exercise experience and group cohesion. So, it is hypothesized that: (1) the motivations of participating in a square dance group can be simply divided into physical fitness and psychological benefits; (2) the prediction of the positive aspect of Subjective Exercise Experience on Group Cohesion is in contrast to that of the negative aspects of Subjective Exercise Experience; (3) there could be a variable, such as years of participation, that plays a role of mediator in the association between Subjective Exercise Experience and Group Cohesion; (4) characteristics of gender cause the motivation differences, which, to some extent, influence the associations of years of participation with Subjective Exercise Experience and Group Cohesion. For testing the above hypothesis, the score of Subjective Exercise Experience Scale was regarded as the predictor (Positive well-being, Psychological fatigue, and Psychological distress), with the score of Group Environment Questionnaire as the dependent variable (Group integration—task, Group integration—social, individual attractions to group—task and Individual attractions to group—social).

## 2. Materials and Methods

### 2.1. Process

This study seriously abided by the operation rules of standard biosecurity and institutional safety. Before the formal questionnaire, we also provided informed consent for the participants. With the help of the person in charge, 1468 female participants from 31 provinces of China (e.g., Northeast: Liaoning, etc., East: Beijing, etc., West: Qinghai, etc., and Center: Hubei, etc.) and abroad separately, were invited to finish online questionnaires from 1 October to 24 October in 2019. The division of the provinces was based on objective factors such as geographical location and economy development [66], to explore various demographic characteristics’ effects on the association between subjective exercise experience and group cohesion. Several choices and completions were designed as in Table 1 below.

### 2.2. Participants

The validation of data was taken into consideration; some samples were deleted due to the limitation of time (>120 s) and age (≥18 years old) during the analysis. In addition, for the purpose of this study, self-reports that noted that he/she had not participated in square dance, or with missing information, were also regarded as invalid. For the reason that the fraction of the remaining data (1238 samples) were submitted by males, 80 responses were then selected using random number generators from the 1164 females (random sampling seed = 1117).

After a preliminary analysis of the descriptive statistics, we conducted an analysis with the rest of the data, containing 130 samples (the index was set as f^2^ = 0.15, α = 0.05, 1 − β = 0.95, and the G*Power 3.1.9.2 calculated total samples was 119 < 130), of males aged from 20 to 76 years old (50.524 ± 14.284) and females aged from 19 to 70 years old (55.403 ± 10.285). Other data are shown in Table 2.

### 2.3. Instrument

#### 2.3.1. Subjective Exercise Experience

The Subjective Exercise Experience measures using the Subjective Exercise Experience Scale (SEES) [67] strongly demonstrated the factory, development, and preliminary validation of three factors; namely, Positive Well-Being (PWB, e.g., I feel great), Psychological Fatigue (PF, e.g., I feel drained), and Positive Distress (PD, e.g., I feel awful), with the data being collected from middle-aged (M = 55 years) exercisers. It also showed a highly reliable result (α_PWB_ = 0.86, α_PF_ = 0.88, α_PD_ = 0.85) for the college students (age M = 20.78 years, SD = 2.18). In this study, Subjective Exercise Experience was measured using 12 items from mentioning three dimensions of the SEES; each dimension contained four items. Additionally, a Chinese version of SEES has been repeatedly confirmed for its good reliability (average α = 0.86) and validation [68]. These three dimensions were scored on a 7-point Likert scale from not at all (1) to very much so (7).

#### 2.3.2. Group Cohesion

Group Cohesion was measured using a Group Environment Questionnaire (GEQ) of 15 items based on a conceptual model that contains four constructs such as Group Integration—Task (GI-T; e.g., Our team is united in trying to reach its goals for performance), Group Integration—Social (GI-S; e.g., Our team would like to spend time together in the off season), Individual Attractions to Group—Task (ATG-T, e.g., I am not happy with the amount of playing time I get), and Individual Attractions to Group—Social (ATG-S; e.g., Some of my best friends are on this team) [69]. Its validation was considered to be good, with the evaluation (α_ATG-T_ = 0.65, α_ATG-S_ = 0.64, α_GI-T_ = 0.71 and αGI-S = 0.72) and reliability also being analyzed with the athletes. The original GEQ was adjusted to Chinese from Hong [70], which was scored on a 7-point Likert scale to indicate the extent of agreement of each item, from extremely agree (7) to extremely disagree (1). The Chinese version of GEQ also has excellent internal consistency (α_ATG-T_ = 0.76, α_ATG-S_ = 0.75, α_GI-T_ = 0.85 and α_GI-S_ = 0.78), test–retest reliability (r_ATG-T_ = 0.70, r_ATG-S_ = 0.80, r_GI-T_ = 0.74 and r_GI-S_ = 0.70), and construct and criterion validity.

### 2.4. Statistic Analysis

The open question, item 22, “What you gained from participating in square dance group?” was analyzed through three steps referred to as Objective Grounded Theory (OGT) [60], including open coding, axial coding, and selective coding, in sequence. As the hypotheses noted, the moderated mediation model was well suited to figure out whether a mediating effect differs according to contexts, groups, or the values of another variable. In other words, the strength of an indirect effect depends on the value of the moderator, resulting in a conditional indirect effect [71]. On account of the characteristics of all mentioned variables, compared to analyzing the moderated mediation via linear regression [72,73,74,75], the product-indicator method [76,77,78] and even the latest Latent Moderate Structural equations method (LMS) [79], Multiple-Group Analysis (MGA), is considered to be a more comprehensive approach, along with Structural Equation Modeling (SEM) in the present study, which were conducted with IBM SPSS version 24.0. [80] and AMOS version 23.0. [81].

In more detail, firstly, this allows one to model both the variability common to a latent variable (i.e., error-free scores), as well as the variability that is not explained by the latent variable (i.e., error). Secondly, SEM allows for the creation of the weighted aggregate variables of the targeted constructs [82,83]. Thirdly, complex models, the flexibility of theory, and prediction-oriented analysis motivate the use of SEMMGA in this study [84]. Fourthly, SEMMGA is a suitable and rational process for examining the theoretical model’s goodness of fit, as well as stability. Fifthly, MGA is the most conservative method for the assessment of differences between the path coefficients between two groups [85], which manifests the effects of moderate variables.

To put these into practice, the default Maximum Likelihood Estimation (MLE) procedures are taken into consideration. A modification index was requested for the chi-squared values, = or >10. We used three goodness-of-fit statistics to compare our Confirmatory Factor Analyses (CFA) models and determined the model fit: (1) Root Mean Square Error of Approximation (RMSEA), (2) Comparative Fit Index (CFI), and (3) Parsimonious Normed Fit Index (PNFI). Note that we also report the chi-squared values (53.911) and degrees of freedom values (11) for comparison purposes [86]. Moreover, the significance test and confidence interval of the index of moderated mediation can be obtained by means of bootstrapping with 2000 samples. The indirect effects of the years of participation on Subjective Exercise Experience and Group Cohesion are considered to be significant when the CI (95%) does not contain the number zero [75]. Power analyses were conducted to determine the minimum sample size needed to detect a medium effect size with α = 0.05 and *p* = 0.95 [87].

## 3. Results

### 3.1. Primary Analysis

Based on the analytic framework of OGT, the responses of item 22 can be summarized as Self-Actualization need (sense of honor and patriotic sentiment), Aesthetics need (artistic accomplishment), Cognition need (knowledge acquisition), Esteem need (sense of identity), Attachment need (social circle), and Safety need (fitness and health), from Maslow’ s hierarchy of needs [88,89,90]. Furthermore, positive well-being was added as the seventh item (see Table 3).

A Repeated Measures ANOVA was performed to explore the gender differences in years of participation for each dimension of Subjective Exercise Experience and Group Cohesion. It presented that there were ratings of ATG-T, ATG-S, and PWB reported by females that were significantly higher than for males (see Table 4).

It shows the Pearson correlations between all variables (see in Table 5). As can be seen, years of participation showed significant correlations with either Group Cohesion or Subjective Exercise Experience. Gender had a strong connection with Subjective Exercise Experience and Group Cohesion, whereas it had a weak connection with years of participation.

### 3.2. Multiple Group Analysis

This model was an excellent fit for the data (χ^2^/df = 1.67, CFI = 0.91, RMSEA = 0.07, PNFI = 0.52). The index was calculated excellently, not only for the males’ data, but also for the females’ data (χ^2^/df = 1.89, CFI = 0.90, RMSEA = 0.10, PNFI = 0.53) (χ^2^/df = 1.48, CFI = 0.91, RMSEA = 0.08, PNFI = 0.50). It was constrained with the measurement weights, structural weights, and measurement residuals, to test the stability of the models between male and female (see Table 6). Only the *p* values were not significant, but also the index of each model was good enough, and the differences between the path coefficients made sense statistically.

**Figure 1:**
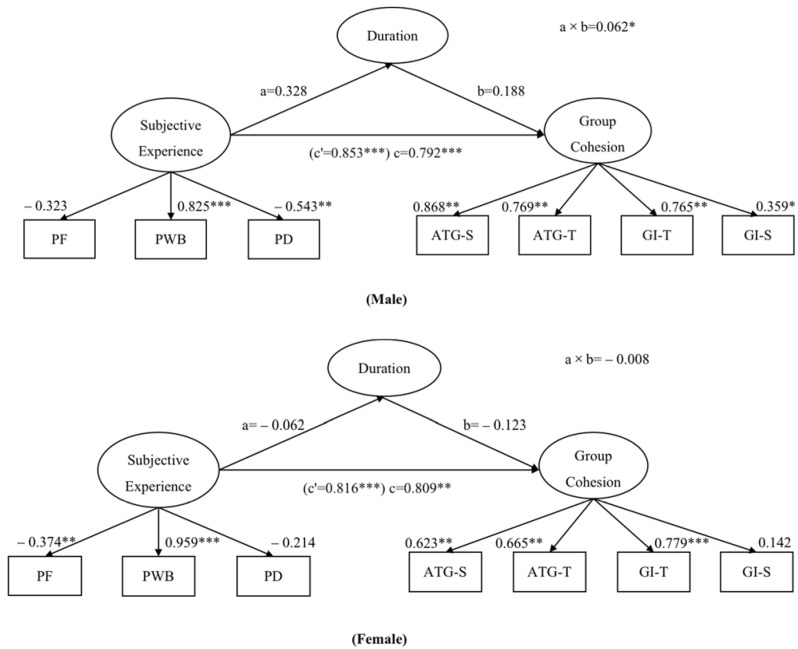
Mediation model for male group (N = 63) and female group (N = 67). Note: PF = Psychological Fatigue, PWB = Positive Well-Being, PD = Psychological Distress, GI-T = Group Integration—Task, GI-S = Group Integration—Social, ATG-T = Individual Attractions to Group—Task, ATG-S = Individual Attractions to Group—Social. Summary standardized path coefficients representing the effects of Subjective Exercise Experience and income rating on Group Cohesion. All values represent standardized estimates/coefficients. *** *p* < 0.001, ** *p* < 0.01, * *p* < 0.05.

Detailed results of the mediation analysis are displayed in Figure 1, where the effect of Subjective Exercise Experience on years of participation is illustrated as a. The effect of years of participation on Group Cohesion is illustrated as b. The indirect effect is the product of paths a and b (ab), and is illustrated as a × b. The direct effect and the total effect are illustrated as c and c’, respectively. Through the bootstrapping procedure, the results indicated that the indirect effect of Subjective Exercise Experience on Group Cohesion through years of participation was only significant with the male group (a × b = 0.062, 95% CI [0.001, 0.181], with standard error (SE) = 0.062, *p* = 0.048). Approximately 75.9% of the variance in Group Cohesion was accounted for by Subjective Exercise Experience and years of participation. The direct effect of Subjective Exercise Experience on Group Cohesion was significant when controlling for years of participation (c = 0.792, 95% CI [0.502, 1.027], SE = 0.140, *p* < 0.001). Additionally, the total effect was significant (c’ = 0.853, 95% CI [0.564, 1.074], SE = 0.135, *p* < 0.001). In summary, the results demonstrated the presence of significant direct and indirect effects in the mediation model for the male group; Subjective Exercise Experience remained a significant predictor of Group Cohesion after including years of participation as a mediator, which suggested that this model was a partial mediation. The female group hardly represented the significant indirect effect of Subjective Exercise Experience on Group Cohesion through years of participation (a × b = 0.008, 95% CI [−0.012, 0.075], SE = 0.023, *p* = 0.268).

An evaluation of the specific paths of the male group from Subjective Exercise Experience to years of participation to Group Cohesion indicated that Subjective Exercise Experience was a significantly positive predictor of Group Cohesion (B = 0.595, β = 0.792, SE = 0.140, *p* < 0.001) and years of participation (B = 0.535, β = 0.328, SE = 0.171, *p* = 0.064), which was a quite significant predictor of Group Cohesion (B = 0.087, β = 0.188, SE = 0.149, *p* = 0.137). This was owing to the factor that gender’s moderating role made differences through two paths in the meantime, both of which changed significantly positive predictions of Subjective Exercise Experience, years of participation, and Group Cohesion into fairly negative ones (B = −0.096, β = −0.067, SE = 0.123, *p* = 0.645) (B = −0.039, β = −0.119, SE = 0.122, *p* = 0.250), whereas it remained a significantly positive prediction of Subjective Exercise Experience and Group Cohesion (B = 0.373, β = 0.785, SE = 0.165, *p* = 0.003). Differences between the path coefficients among the male and female groups showed significant differences for the associations of Subjective Exercise Experience and years of participation (t = −2.043), years of participation, and Group Cohesion (t = −1.962).

## 4. Discussion

The purpose of this study was to assess the relationship between positive and negative, with the two opposite aspects of subjective exercise experience and group cohesion in square dance participants, and to investigate whether years of participation acts as a mediating role in this relationship, which is moderated by gender. Therefore, this study contributes to the research by adding the information about the relationships between all these four variables. Moreover, the motivation of participating in square dance can be divided into physical fitness and psychological benefits, including Self-Actualization, Aesthetics, Cognition, Esteem, Attachment, Safety, and Positive.

In a sense, shared identity amongst participants may encourage participants to view others as a source of social support, which further contributes to a sense of health and well-being [38], expressing that Subjective Exercise Experience had a significantly positive correlation with Group Cohesion among the male and female groups. However, some consequences may be negative, and this same sense of shared identity may result in a loss of disgust at the prospect of sharing resources [91]. For a beneficial role of social cohesion in maintaining or increasing needs satisfaction [92], the positive aspect of Subjective Exercise Experience had a significant positive correlation with Group Cohesion, while negative aspects of Subjective Exercise Experience had a negative one, supporting most previous studies.

More specifically, PWB, the positive aspect of Subjective Exercise Experience, will be greater, with more years of participation substantially increasing this factor among males [93], though this was completely reversed in the situation of females [94]. There were opposite outcomes for associations between years of participation and the negative aspects of Subjective Exercise Experience, PD and PF. The ratings of PD were less, with years of participation rapidly increasing in males, and with years of participation decreasing in females [95]. As for the ratings of PF, the opposite occurred [96]. In light of these results, different patterns of subjective exercise experience within high PWB, and low PD and PF, can link to a high degree of group cohesion in the male and female groups, which means that a development of fitness activity should be based on an appropriate training intensity and content [97], through before- and after-scorings of specific scores before and after SEES, based on an 8-week jazz dance class (twice a week, at 70 min per session) [98]. In the female group, there was a small decrease between PWB and PD, while this was the opposite in the male group. PF shows both reductions in the female group and the male group. Task aspects of Group Cohesion consisted of ATG-T and GI-T, which were defined by Carron et al. [69] as a general orientation toward achieving the group’s goals and objectives. It gave information concerning that the ratings of task aspects of Group Cohesion, ATG-T, and GI-T, will be much more with years of participation increasing among males and decreasing among females [36]. The social aspects of Group Cohesion consisted of ATG-S and GI-S, which were defined by Carron et al. [69] as a general orientation toward developing and maintaining social relationships within the group. It was among females that the association between ratings of ATG-S were strongly negative, and that the association between ratings of GI-S was negative. When it came to males, an increase of duration had strong association with the ratings of ATG-S [53] and the ratings of GI-S. These were not completely expected, because a few previous studies had proven it indirectly. Group physical activity was prospectively associated with a lower degree of psychological distress and a higher degree of well-being among men, but not among females [99], and the advantage of women compared with men with regard to daily life accounted for gender differences in psychological distress [94,100]. Furthermore, numerous subsequent psychological well-being and psychological distress outcomes (i.e., higher positive affect and lower negative affect) were associated with perceived social cohesion over the four-year period [101]. From this point of view, increasing the years of participation will lead to a high social aspect of Group Cohesion, which is consistent with males but contrary to females.

This is due to the six key themes that were identified from the qualitative studies as being important for adherence to group exercise programs: social connectedness, participant perceived benefits, program design, empowering effects, and instructor and individual behavior [102]. Gender does cause the extent to which Subjective Exercise Experience affects Group Cohesion and the effect of pathways mediated by years of participation varies, which is in line with the findings of previous studies, where males over 18 years old in Busan showed higher scores with regard to social behavior with external displays than females [103]. This is consistent with the ability of years of participation to influence participants’ subjective perceptions of exercise. Furthermore, cohesion might have a differential effect on an individual’s decision to regularly participate in exercise, depending on the duration of membership in the group [53]. The current study was unexpected, compared to a previous study among 301 male futsal athletes that revealed that the satisfaction of basic psychological needs presented a significant association with group cohesion according to GEQ, explaining 28% of Task Cohesion and 63% of Social Cohesion variances. The satisfaction of the three basic psychological needs is associated with Task Cohesion, while only the satisfaction of the basic psychological needs of Relatedness and Competence, instead of Autonomy, was associated with Social Cohesion [104]. This may be due to the years of participation that added a further explanation to task cohesion; hence, the variance of social cohesion, other than variance of task cohesion, could be predicted to a greater extent. From this point of view, the best interpretation of fitness increase (35% in Irishmen under 35 years of age) [105] were in terms of endurance. The respondents referred mainly to endurance in the context of exercise activity; for example; “I am no longer getting a sore back”. For some, endurance was interpreted in the context of everyday activities. One stated, for example, that “It makes me feel fresh and energetic for the whole day”. This could be easily understood that the females who still held onto exercising experienced a negative state. On the other hand, females’ thwarting behaviors always did much damage to their weaker intentions to exercise in the future, but young male adults and more experienced exercisers had stronger intentions towards exercising [106]. Furthermore, an investigation into the self-reported frequency, intensity, and duration of anger that they felt during tennis matches, and perceived effects on the performance of 180 (92 males) recreational tennis players showed no gender differences in subjective experience of anger, whereas gender differences did emerge in its regulation strategies and expression [84]. Not only that, according to the flow experience in a study of 1094 Spanish adolescents (12~18 years old), the results indicated that intrinsic motives predicted physical practice, whilst extrinsic motives could not [107]. It pointed out that promoting enjoyment-related motives and positive subjective experiences such as flow, which corresponded to autonomy, might help in enhancing adherence to exercise.

Nevertheless, several limitations of this study should be mentioned. Primarily, this study was based on a moderate exercise form, and the indicators that represented the effects of square dance could be denied with both subjective and objective measurements. Furthermore, only a cross-sectional inquiry was conducted, and a long-term follow-up of subjects was lacking. Future studies should conduct a longitudinal analysis to figure out the changes of Subjective Exercise Experience, Group Cohesion, and other geographical information caused by “square dance”, and how long the improvements maintain. Moreover, for providing a better guidance for national fitness, future studies should focus on exploring a variety of factors associated with the time of participation, including asking about physical activities for meeting people’s needs. By virtue of policy [108,109], the item inquiring about occupation did not make sense at all. Thus, there was no detailed discussion of the subjects’ occupational profiles, ignoring the possible influence of occupation on the square dance participant population. Future researchers can also investigate the patterns of the relationship between the subjective exercise experience and group cohesion that would be optimal for the male and female groups, respectively, to enrich the repository of the research field. Future studies should be more concerned and sensitive toward other characteristic differences in terms of the subjective exercise experience in relation to group cohesion, such as educational background, income level, and social and cultural differences [39,110,111]. Finally, the study has limitations in the selection of sample size, in order to prove the comprehensive, specific, and accurate effects of square dance on mental and physical health caused by different motivations, and the selection of participants for such a study should be made over a larger scale.

## 5. Conclusions

By comparing the differences between male and female groups in terms of years of participation, this study has found that duration should be the partial mediator of the relationship between subjective exercise experience and group cohesion. These empirical findings contributed to the participants of square dance, bridging the gap between years of taking part in square dance for men’s subjective exercise experience and group cohesion research, and providing a certain theoretical basis and practical implications for the better development of square dance as a sport to understand the different mechanisms of males and females underlying group exercising behavior, as an approach for constructing a public health system [112,113,114]. Not only did such a mediator model demonstrate associations among group cohesion, as well as the length of duration and subjective experience among different genders within a filed raising physical activity for Health China [115], but additionally, during the COVID-19 pandemic, dance-based mind–motor activities such as folk dance have been suggested as a form of physical exercise with extended benefits beyond physical health, which could advantage the exercisers’ cognition, social interaction, quality of life, and motivation to be physically active. It has the characteristics of a good analgesic effect, improvement in exercise ability, no side effects, convenience of operation, a low economic burden, and higher conduciveness toward promoting the rehabilitation of patients [116].

Great efforts could be directed toward certain groups, rather than just toward individuals, to help with public fitness development for the interaction of mental health and cohesion in society [117]. Therefore, future studies should focus on a particular characteristic or a further mechanism in examining the influence of subjective exercise experience on group cohesion, so that the relationship is more applicable and effective.

## Figures and Tables

**Table 1:** Self-edited list of questions.

Question Type	Contents
Choices	How much money do you will earn every month?
	What is your highest educational level?
	What is your gender?
Completions	When did you begin to participate in the square dance group?
	What have you gained from participating in the square dance group?
	What is your job?
	How old are you?

**Table 2:** Summary of the demographic information (N = 63 + 67).

Demographic Measures		Number of Males	Number of Females	Demographic Measures		Number of Males	Number of Females
Education	Elementary	0	0	Income level	≤2000	9	16
	Junior high	12	15		2001~3500	10	23
	Senior high	14	29		3501~5000	20	16
	Undergraduate	31	22		5001~6500	10	6
	Postgraduate	6	1		6501~8000	6	2
					>8000	8	4

**Table 3:** The answers of question 22 to measure the motivation of participating in square dance (N = 63 + 67).

Hierarchy of Needs		The Number of Males	The Number of Females
Self-Actualization	e.g., I took pride in my or our team’s achievement. I experienced deeper patriotism.	1	0
Aesthetics	e.g., I cultivated my artistic aesthetics.	2	3
Cognition	e.g., I have been in command of dance skills gradually. I learned more information from others.	3	8
Esteem	e.g., I became more confident with performance.	1	0
Attachment	e.g., I made more friends. Since retirement, I finally felt a sense of belonging.	10	15
Safety	e.g., I built up my body.	37	32
Positive	e.g., I experienced pleasure/well-being.	28	38

**Table 4:** Results of Repeated Measures ANOVA in different genders (N = 63 + 67).

Variables	Male (n = 63) M ± SD	Female (n = 67) M ± SD	F (1, 62)	*p*
Years of participation	4.08 ± 5.14	4.31 ± 5.12	0.02	0.881
ATG-T	16.83 ± 2.37	17.82 ± 1.60	8.77	0.004
ATG-S	23.13 ± 3.27	24.75 ± 1.93	10.74	0.002
GI-T	23.38 ± 3.09	24.25 ± 2.15	4.94	0.030
GI-S	19.16 ± 3.04	20.06 ± 2.97	3.23	0.077
PWB	20.30 ± 3.81	22.10 ± 3.79	7.42	0.008
PF	12.22 ± 3.98	11.12 ± 8.68	2.57	0.114
PD	8.68 ± 2.75	8.24 ± 2.66	2.76	0.101

Note: ATG-T = Individual Attractions To Group—Task, ATG-S = Individual Attractions To Group—Social, GI-T = Group Integration—Task, GI-S = Group Integration—Social, PWB = Positive Well Being, PF = Psychological Fatigue, PD = Psychological Distress.

**Table 5:** Pearson correlations between demographic information, Group Cohesion, and Subjective Exercise Experience (N = 63 + 67).

Variables	1	2	3	4	5	6	7
1. Age	1.000						
2. Income	0.057	1.000					
3. Education	−0.229 **	0.485 **	1.000				
4. Years of participation	0.253 **	0.063	−0.072	1.000			
5. Gender	0.190 *	−0.258 **	−0.207 *	0.022	1.000		
6. Group Cohesion	0.217 *	−0.116	−0.215 *	0.204 *	0.291 **	1.000	
7. Subjective Exercise Experience	−0.034	−0.099	−0.021	0.213 *	0.021	0.142	1.000

Note: All values represent standardized estimates/coefficients. ** *p* < 0.01, * *p* < 0.05.

**Table 6:** Comparison of models index.

Model	Δχ^2^	∆df	*p*	CFI	RMSEA	AIC	PNFI
Measurement weights	11.83	6	0.07	0.88	0.07	164.37	0.57
Structural weights	3.68	2	0.16	0.83	0.08	168.76	0.64
Measurement residuals	11.94	8	0.15	0.78	0.08	172.84	0.67

## Data Availability

All data are available within this manuscript.
